# Ankylosing Spondylitis Presenting as a Severe Kyphotic Deformity Without Pain: A Case Report and Literature Review

**DOI:** 10.7759/cureus.110310

**Published:** 2026-06-05

**Authors:** Hajar Fahli, Elgmiri Hajar, Sara Skalli, Samia Karkouri

**Affiliations:** 1 Physical Medicine and Rehabilitation, Ibn Sina University Hospital, Faculty of Medicine and Pharmacy of Rabat, Mohammed V University, Rabat, MAR

**Keywords:** ankylosing spondylitis, painless presentation, physical medicine, severe kyphosis, spinal deformity

## Abstract

Ankylosing spondylitis (AS) is a chronic inflammatory disease predominantly affecting the axial skeleton and sacroiliac joints. Progressive structural remodeling may lead to syndesmophyte formation, spinal ankylosis, and the characteristic “bamboo spine” appearance. In advanced stages, spinal deformities and functional impairment may occur.

We report the case of a 32-year-old male presenting with progressive spinal deformity and cosmetic concern without significant current pain. The deformity had progressively developed since adolescence, beginning at the age of 15, and was initially associated with alternating hip pain. Clinical examination revealed marked cervicothoracic hyperkyphosis, sagittal imbalance, left shoulder elevation, and right pelvic tilt. Spinal mobility assessment demonstrated severe limitation, with a Schober index of 1 cm and thoracic expansion reduced to 4 cm. Imaging studies revealed a bamboo spine appearance with fusion of the posterior vertebral elements, while pelvic radiography demonstrated bilateral sacroiliac ankylosis. Laboratory investigations confirmed HLA-B27 positivity. Pulmonary function tests showed a restrictive ventilatory pattern. Bath Ankylosing Spondylitis Disease Activity Index (BASDAI) and Bath Ankylosing Spondylitis Functional Index (BASFI) scores indicated clinically significant disease activity and moderate functional impairment, respectively. The patient was enrolled in a multidisciplinary management program including motor and respiratory rehabilitation, rheumatological follow-up, and referral for surgical evaluation of the fixed kyphotic deformity.

This case highlights an advanced presentation of AS presenting primarily with spinal deformity rather than active pain. It emphasizes the importance of early recognition and regular assessment of spinal mobility in order to prevent irreversible structural damage and long-term functional impairment.

## Introduction

Ankylosing spondylitis (AS) is a chronic inflammatory rheumatic disease predominantly affecting the sacroiliac joints and the axial skeleton. The disease usually begins in early adulthood and is strongly associated with the HLA-B27 genetic marker. Its prevalence varies worldwide, ranging from approximately 0.1% to 1.4%, depending on the studied population and the prevalence of HLA-B27.

The inflammatory process primarily involves the entheses and may lead to progressive structural remodeling of the spine. Recurrent cycles of inflammation and repair promote syndesmophyte formation and ossification of spinal ligaments, eventually resulting in vertebral ankylosis and the characteristic radiological appearance known as a “bamboo spine”.

In advanced stages, AS may lead to severe structural spinal deformities, including thoracic hyperkyphosis, loss of lumbar lordosis, and marked restriction of spinal mobility. These alterations can result in sagittal imbalance, postural abnormalities, and respiratory impairment due to reduced thoracic cage mobility.

We report the case of a 32-year-old man presenting with advanced AS and severe cervicothoracic hyperkyphosis, in whom postural concern constituted the main reason for medical consultation.

## Case presentation

A 32-year-old man presented for medical evaluation of a progressive spinal deformity associated with postural concerns. The patient reported that the deformity had gradually developed since the age of 15 years and was initially associated with alternating hip pain; however, no medical attention was sought at that time. Although the deformity was initially associated with alternating hip pain during adolescence, the patient no longer reported significant spinal or hip pain at presentation, despite marked structural deformity and mobility limitation. His main concern was the progressive postural deformity. No history of extra-articular manifestations, including uveitis, psoriasis, or inflammatory bowel disease, was noted.

Physical examination showed preserved active and passive range of motion in both the upper and lower limbs. Muscle strength was normal (5/5) in all extremities, with preserved muscle tone. Neurological examination revealed no sensory deficit, with intact superficial and deep sensation.

Postural evaluation demonstrated asymmetry of the shoulder girdle, with elevation of the left shoulder associated with right pelvic tilt. Palpation of the spine revealed paraspinal muscle spasm without localized tenderness.

Spinal mobility assessment demonstrated severe restriction of lumbar movement. Lumbar extension was impossible because of the fixed kyphotic deformity, lateral flexion was absent bilaterally (0 cm), and axial rotation was markedly limited. Frontal plane evaluation showed a deviation of the S2 plumb line of 3 cm toward the right, while the tragus-acromion distance measured 10 cm bilaterally.

Sagittal plane assessment revealed significant sagittal imbalance, with an occiput-to-wall distance of 19 cm, a C7-to-wall distance of 17 cm, and a D6-to-wall distance of 9 cm. The L3-to-wall distance measured 1 cm to the left, and the S2-to-wall distance measured 1.5 cm. Cervical mobility assessment revealed a chin-to-sternum distance of 20 cm in extension and 0 cm in flexion.

Transverse plane evaluation revealed trunk rotation abnormalities, with gibbosity measuring 3° in the cervical region, 8° in the dorsolumbar region, and 12° in the lumbar region toward the left. The chin-to-acromion distance measured 13 cm on the right and 15 cm on the left. Thoracic expansion was reduced to 4 cm, and the Schober index measured 1 cm, indicating severe limitation of lumbar flexion. The finger-to-floor distance was 12 cm, reflecting reduced flexibility. 

Laboratory investigations revealed an erythrocyte sedimentation rate (ESR) of 12 mm/h and a C-reactive protein (CRP) level of 5 mg/L, indicating the absence of significant systemic inflammatory activity at the time of evaluation. HLA-B27 testing was positive. Disease activity and functional impairment, assessed using the Bath Ankylosing Spondylitis Disease Activity Index (BASDAI) and the Bath Ankylosing Spondylitis Functional Index (BASFI), were both scored at 4, indicating clinically significant disease activity and moderate functional impairment.

**Figure 1:**
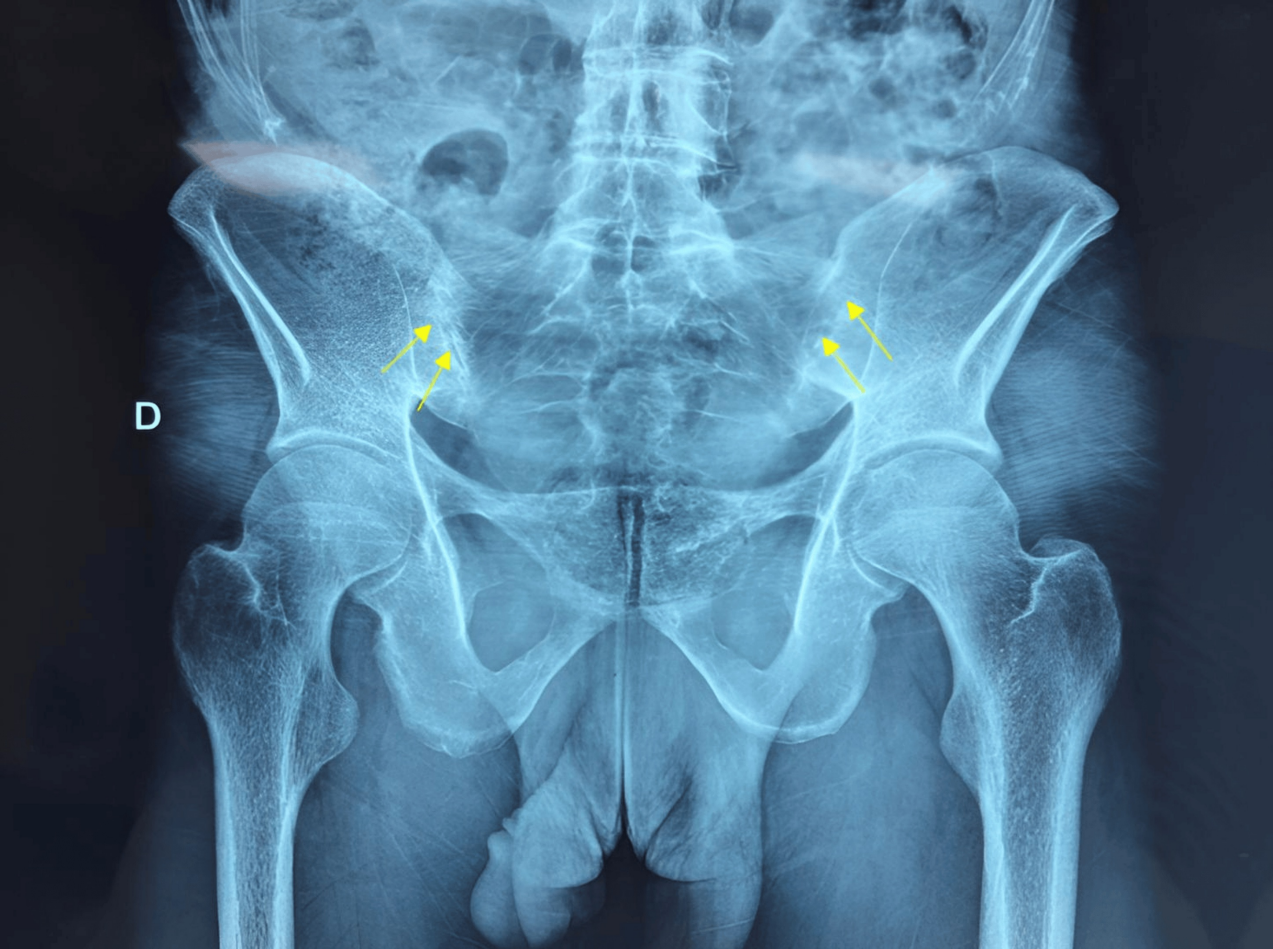
Anteroposterior view of the pelvis demonstrating bilateral sacroiliac joint ankylosis with joint space obliteration, consistent with advanced ankylosing spondylitis (arrows).

**Figure 2:**
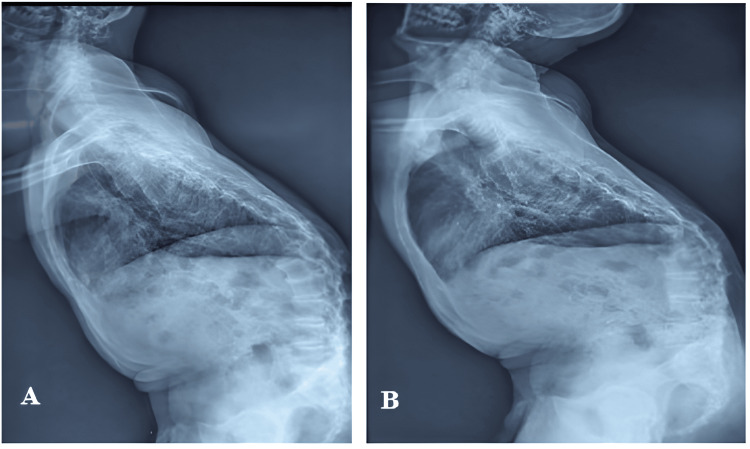
Lateral standing spine radiographs demonstrating severe thoracolumbar kyphosis in advanced ankylosing spondylitis. (A) Without postural correction. (B) Attempted postural correction showing minimal improvement, consistent with a fixed deformity.

**Figure 3:**
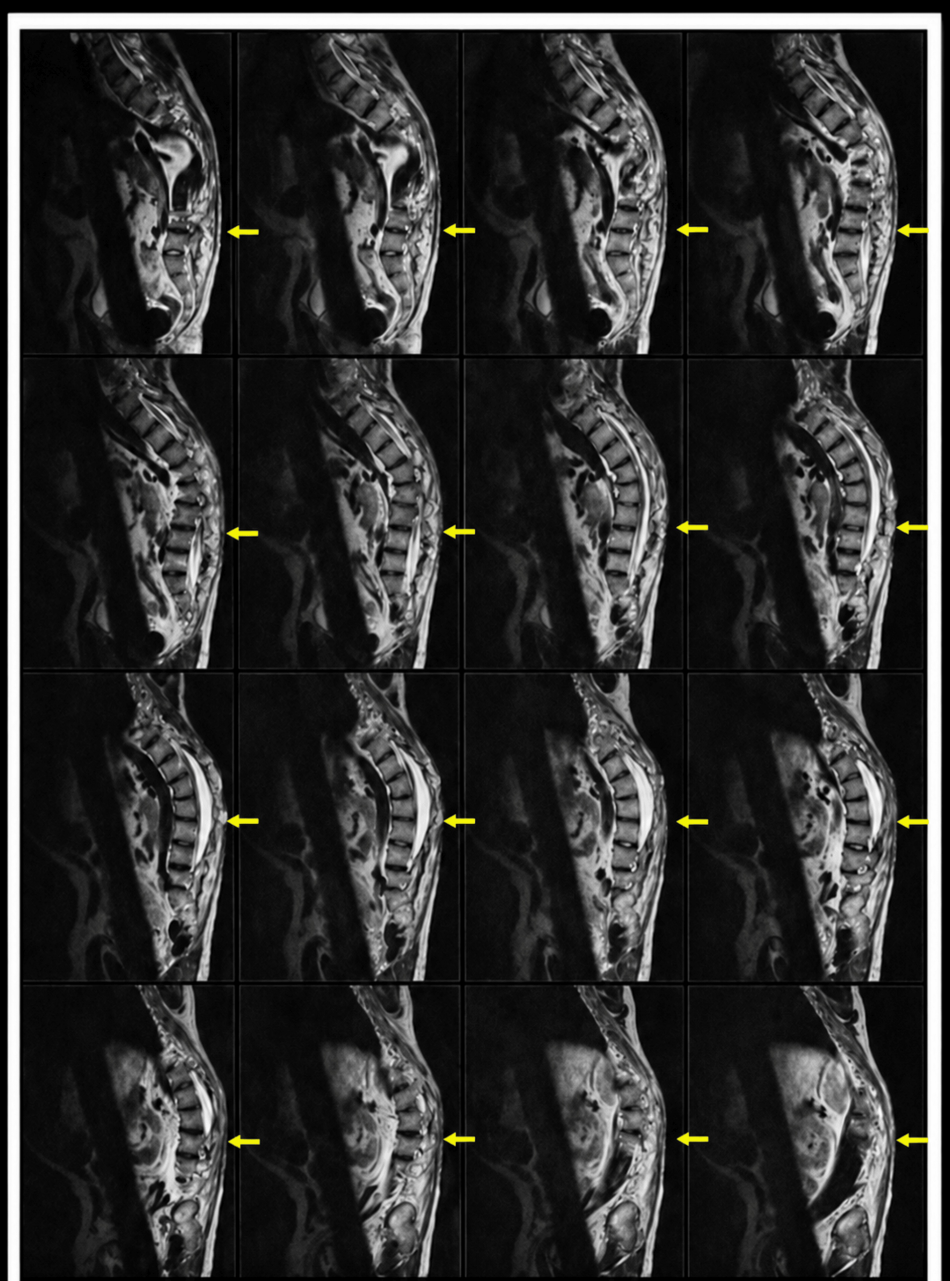
Sagittal CT images of the spine demonstrating extensive ankylosis with fusion of the posterior vertebral elements in advanced ankylosing spondylitis (arrows).

**Figure 4:**
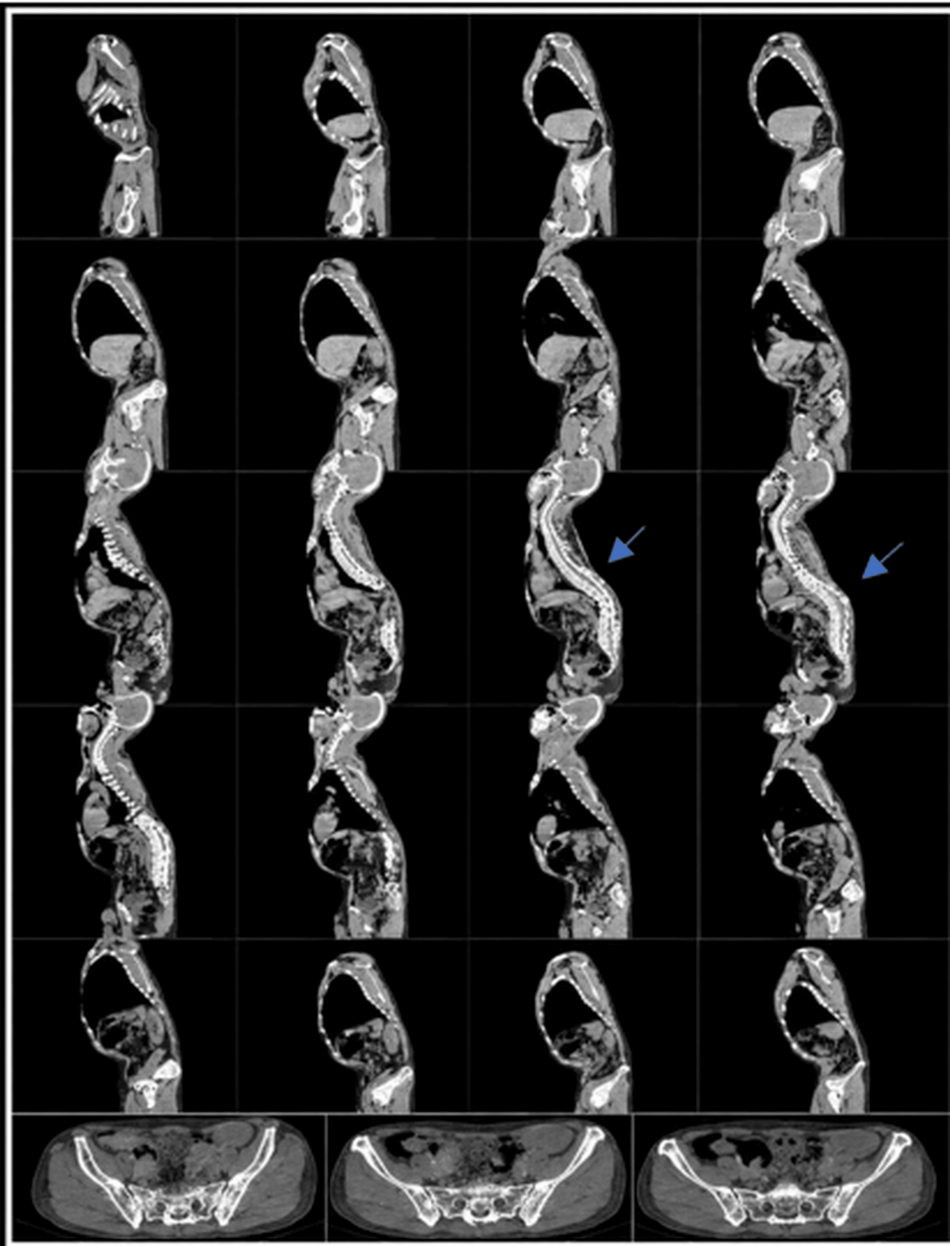
Whole-spine sagittal MRI images and axial pelvic MRI sections demonstrating advanced ankylosing spondylitis with extensive syndesmophyte formation, bamboo spine appearance, and fusion of the posterior vertebral elements (arrows).

Imaging studies demonstrated advanced structural involvement of the spine. Plain radiographs of the spine and pelvis revealed syndesmophyte formation with a characteristic “bamboo spine” appearance, associated with bilateral sacroiliitis and sacroiliac joint ankylosis. Lateral standing radiographs demonstrated severe fixed thoracolumbar kyphosis with loss of physiological lumbar lordosis and marked anterior trunk displacement, indicating global sagittal imbalance, without evidence of fracture or focal bone lesion (Figures 1-2). Computed tomography confirmed fusion of the posterior vertebral elements (Figure 3), while magnetic resonance imaging demonstrated fusion of the posterior vertebral arches (Figure 4).

**Figure 5:**
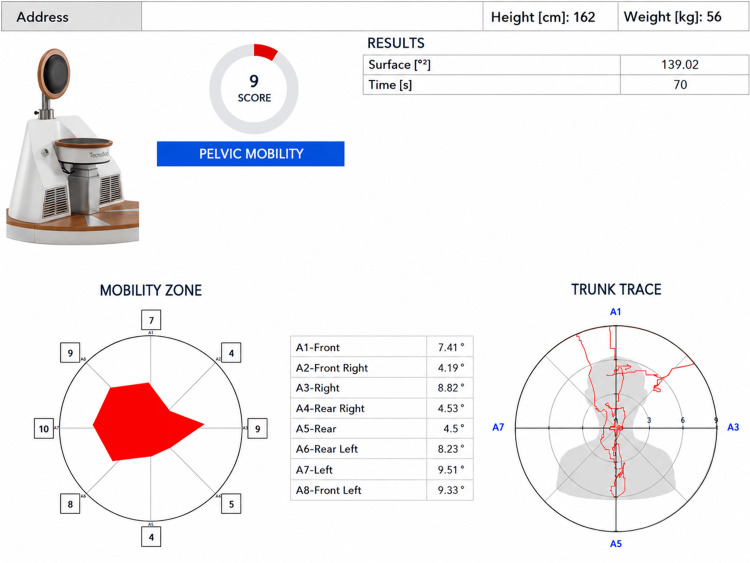
Trunk mobility assessment showing reduced range of motion and altered movement pattern in a patient with ankylosing spondylitis. This image was not generated using AI; it was exported directly from the ProKin 252® system software (TecnoBody, Bergamo, Italy).

Pulmonary function tests demonstrated a restrictive ventilatory pattern. Seated posturographic assessment using the Prokin 252® system (TecnoBody, Bergamo, Italy) revealed reduced pelvic mobility, suggesting impaired lumbopelvic dissociation. Standing posturographic assessment performed with eyes open and closed revealed impaired static balance with increased postural sway, reflecting altered postural control related to severe axial deformity (Figure 5).

**Figure 6:**
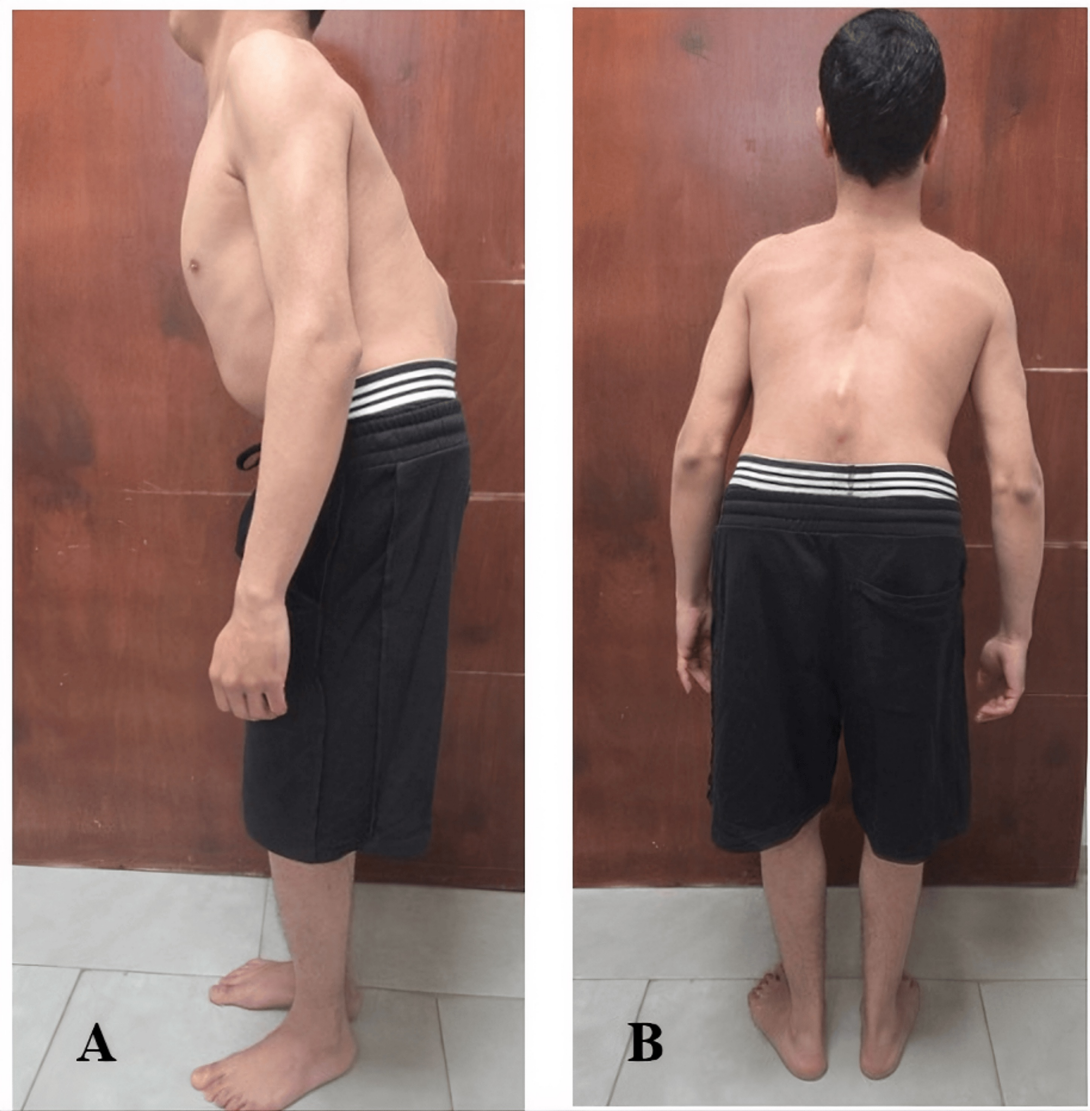
(A) Sagittal spinal deformity with forward trunk flexion revealing ankylosing spondylitis. (B) Posterior view showing forward trunk flexion and prominent spinous processes without coronal deformity.

Clinical photographs illustrated marked cervicothoracic hyperkyphosis associated with forward trunk inclination and postural imbalance (Figure 6).

The patient was referred for multidisciplinary management. A rehabilitation program was initiated, including motor physiotherapy aimed at maintaining spinal mobility, improving postural alignment, and preserving functional capacity, as well as respiratory physiotherapy to address the restrictive ventilatory impairment associated with reduced thoracic expansion. The patient was also referred for regular rheumatological follow-up for ongoing disease assessment and therapeutic management. Given the severity of the fixed kyphotic deformity, marked sagittal imbalance, and advanced structural damage, a spine surgical consultation was requested to evaluate the feasibility and potential benefits of corrective osteotomy. At the time of writing, the patient remained under multidisciplinary follow-up.

## Discussion

The present case illustrates an unusual presentation of advanced AS in which severe cervicothoracic kyphosis and sagittal imbalance, rather than persistent pain, constituted the main reason for seeking medical attention. Although inflammatory symptoms were present during adolescence, pain was no longer a prominent complaint at the time of evaluation. Similar presentations have been reported in advanced disease, where fixed deformities and functional limitations may overshadow inflammatory symptoms and become the predominant clinical features [1].

The most likely explanation for this presentation is a long-standing inflammatory disease that remained unrecognized and progressively evolved toward irreversible structural damage. Previous longitudinal studies have shown that cumulative inflammatory burden plays a major role in structural progression in AS, promoting syndesmophyte formation, ligament ossification, and eventual ankylosis [2]. In advanced stages, functional impairment and spinal mobility limitation become increasingly related to structural damage rather than active inflammation [3]. This concept is particularly relevant in our patient, whose fixed thoracolumbar kyphosis, bamboo spine appearance, sacroiliac ankylosis, and markedly reduced spinal mobility suggest a predominantly structure-driven disease state.

Another important aspect highlighted by this case is the dissociation between structural severity and current inflammatory activity. Normal CRP and ESR levels do not exclude active disease activity, as these markers have limited sensitivity and may remain normal in a substantial proportion of patients with axial spondyloarthritis [4]. Nevertheless, in the absence of significant inflammatory pain and with imaging findings dominated by ankylosis rather than active inflammatory lesions, the clinical presentation in this patient appears more consistent with relatively low inflammatory activity burden and advanced irreversible structural damage. This observation may reflect the transition from an inflammation-dominant phase to a structure-dominant phase of the disease.

The restrictive ventilatory impairment observed in our patient further supports this interpretation. In AS, respiratory dysfunction is mainly related to reduced chest wall mobility secondary to involvement of the costovertebral and costosternal joints, as well as spinal deformity. Previous studies have shown that pulmonary restriction correlates more strongly with spinal stiffness and reduced thoracic expansion than with disease activity indices [5]. In patients with advanced kyphotic deformity, thoracic cage rigidity and altered biomechanics may significantly impair respiratory function [6]. In this context, the restrictive pattern observed in our patient is more likely related to advanced structural involvement than to active inflammatory disease.

Another key feature of this case is the marked diagnostic delay. Symptom onset during adolescence, followed by diagnosis only in adulthood, reflects a prolonged disease course. Previous systematic reviews have reported an average diagnostic delay of approximately six to seven years in axial spondyloarthritis, although substantially longer delays may occur [7]. Delayed diagnosis has been associated with increased structural damage, functional impairment, and worse long-term clinical outcomes. In the present case, the prolonged untreated period likely contributed significantly to the development of severe spinal deformity and functional impairment.

This case also illustrates an atypical clinical presentation of advanced AS, in which pain is no longer the predominant symptom despite severe structural involvement. This pattern may reflect an advanced ankylosed stage of the disease, in which inflammatory activity becomes less prominent while mechanical stiffness and fixed deformities predominate [8].

From a therapeutic perspective, this case underscores the importance of early recognition and multidisciplinary management. Current ASAS-EULAR recommendations emphasize the combined role of non-pharmacological and pharmacological strategies, including exercise therapy and biologic treatment when indicated [9]. Early intervention remains essential to limit irreversible structural damage and preserve long-term functional capacity.

Overall, this observation emphasizes that severe structural disease may exist even in the absence of marked inflammatory symptoms [10]. Clinicians should therefore maintain a high index of suspicion for axial spondyloarthritis in patients presenting with marked postural abnormalities, spinal stiffness, reduced chest expansion, and/or restrictive ventilatory impairment, even when inflammatory pain is minimal. Early diagnosis and appropriate management remain essential to prevent irreversible structural damage and long-term disability [11].

## Conclusions

This case illustrates the severe structural consequences of delayed diagnosis and long-standing, untreated AS. Marked cervicothoracic hyperkyphosis, profound restriction of spinal mobility, reduced chest expansion, and the characteristic bamboo spine appearance reflect advanced structural damage and its functional repercussions. Despite the absence of significant pain at the time of evaluation, the patient exhibited extensive radiographic abnormalities, severe postural deformity, respiratory impairment, and marked limitations in spinal mobility. These findings highlight that advanced AS may be dominated by structural and functional consequences rather than active inflammatory symptoms.

This observation underscores the importance of early recognition of axial symptoms and regular clinical assessment in young patients with progressive postural abnormalities. Early diagnosis and appropriate multidisciplinary management are essential to prevent irreversible spinal deformities, respiratory impairment, and long-term functional disability associated with advanced disease.
